# Notes and News

**Published:** 1889-08-24

**Authors:** 


### Notes and News.

Collected and Communicated.

A female inmate of Liverpool Workhouse Asylum lately committed suicide in the most determined manner. She rushed to the fire (why was there no guard?) and set her clothes alight. Several attendants were burnt in trying to rescue her, but her injuries were so great she died in a few hours.

Chinese must be tiresome patients. A weekly paper states that as the native practitioners furnish very large doses, John is apt to think that the foreigner has been mean in giving him such a small quantity, and so takes two or three doses at once. One woman recently gave her infant the ointment which was made up for external application; happily, the child was none the worse.

A debate has taken place at Simla on the alarming result in India of the suspension of the Contagious Diseases Act. Sir Frederick Roberts, the Commander-in-Chief, stated that the percentage of soldiers on the sick list had doubled since the suspension of the Act. The Marquis of Lansdowne pointed out that the Indian Government was bound by the House of Commons resolution. His Excellency hoped, however, that, without overstepping the limits of that resolution, the present Bill would check disease, and that public opinion would support the reasonable steps which were being taken by the Indian Government.

A Damon and Pythias story in a women's paper is amusing. A young fellow is dying of hydrophobia, and promises his comrade not to bite him. At the last, however, the patient so far forgets himself as to clasp his comrade in his arms. "No evil results have yet followed," concludes the narrator, who evidently believes hydrophobia to be infectious. At Bucharest a surgeon is being treated who positively was bitten by a hydrophobia patient, but it is hoped no danger will follow. Four years ago a gamekeeper of Lord Egmont's, when dying of hydrophobia, bit a man, but the disease was not communicated.

Lewes Infirmary continues to do good work in its enlarged wards. Over 1,000 patients were treated last year at a cost of £513. One wonders where they all came from in such a sleepy hollow as Lewes, but perhaps the fifth of November accounts for a good number. More subscribers are needed.

Liverpool Hospital Fund, which amounts this year to £10,000, has been awarded as follows: the Royal Infirmary receives £2,242 10s., the Royal Southern Hospital £1,560, the Northern Hospital £1,365, Dispensaries and District Nursing Society £682 10s. each, Infirmary for Children £585, Stanley Hospital and Eye and Ear Infirmary £487 10s. each. The remainder is divided between a number of smaller institutions.

The Isle of Man Hospital issues its annual report: The number of in-patients for the year is 225 as compared with 181 for the previous year, while the housekeeping bill, including the salaries of matron, nurses, and servants, amounts to £867 11s. 9d., as compared with £589 12s. 3d. for the previous year. The committee wish the friends of the institution to rise to the occasion; and they expect a corresponding increase in the funds put at their disposal. Instead of that, however, they regret to find a falling off to the amount of £75 in the contributions from various sources during the past as compared with the previous year; and they find £76 19s. 11d. due to the bank. It will be a disgrace to the island if the new hospital, built by the munificence of the late Mrs. Noble, is not supported by the people.

Robert Snasshall, in the employ of the London, Brighton, and South Coast Railway Company, has died at Croydon from lockjaw, caused by his thumb being pricked by a nail. Edward Wilkins, aged twenty-three, has died at Hampton Wick from lockjaw. On Saturday, August 3, he was in a yard working at a boat. As he was walking round the bow of the boat he trod on a nail with his left foot, but did not then complain. At tea-time he bathed it in warm water, and thought it was all right. Early the following week, however, it pained him, and he was medically attended. On Wednesday, symptoms of lockjaw set in, and, notwithstanding his being attended by several medical men, including Sir Hugh Bengo, Mr. Jacobson, and Dr. Farr-White, he succumbed on Friday morning, after suffering intensely. It was given in evidence that the nail upon which deceased trod perforated his boot, and caused a small black mark in his foot.

Last Friday afternoon Mr. W. A. Tyssen Amherst, M.P., presented certificates on behalf of the St. John Ambulance Association to 146 police officers, in the theatre of the Royal United Institution. The certificates presented on this occasion brought the total of police officers certificated by the association in the metropolis to 2,231, and the aggregate number of certificates granted to date to nearly 170,000. Sir H. C. Perrott, chief secretary, having remarked that the reports of the examiners were very satisfactory, Mr. Tyssen Amherst presented certificates to police officers from the following stations: Scotland Yard, Kennington-lane, Albany-street, Blackheath-road, Twickenham, and Leman-street. Mr. Mackellar, chief surgeon to the Metropolitan police, testified to the great number of instances which had come under his notice of lives being saved by certificated policemen. The Chairman gave similar evidence with respect to the provinces, and spoke of the great progress of the association's work of recent years. Sir C. Daubeney, G.C.B.; Deputy Inspector-General Coates, R.N.; Dr. Simmonds; Dr. Tayler, and Inspector Peters, A Division, also spoke. Viscount Templetown, G.C.B., Colonel Upton Prior, Major Dalton, R.A., and Mr. J. H. Easterbrook were present.

The following vacancies are announced this week, the amount of the salary in each case being given after the name of the institution:

**Resident Medical Officers.**

Swansea Hospital. Medical Officer.

Birmingham City Asylum. Clinical Assistant.

Cancer Hospital, Fulham-road. Two House Surgeons.—£50, board, &c.

Wallasey Dispensary. House Surgeon.—£110, rooms, &c.

Wolverhampton Hospital. House Physician. — £100, board, &c.

Glasgow Victoria Infirmary. Medical Superintendent.—£125, board, &c.

Birmingham Children's Hospital. Medical Officer and Assistant Medical Officer. — £80 and £40, with board, &c.

Birmingham General Hospital. Surgical Officer.—£130, board, &c.

**Non-Resident Medical Officers.**

Kingston Union. District Medical Officer.—£80.

The hospital to be erected by the Salvation Army, though chiefly in connection with the rescue work, will be open to all, and there will be one or two private wards for paying patients. A lady M.D. has offered to be house physician, and a certificated midwife will have charge of the maternity ward. It is hoped to secure funds enough to keep from twenty to thirty beds going, which Mrs. Bramwell Booth estimates will cost £500 a year.

Earl Percy visited North Shields on August 3 for the purpose of opening the Tynemouth Jubilee Infirmary, the foundation-stone of which, as its name implies, was laid on Jubilee day. The building is of red brick, and is built from Mr. F. R. N. Haswell's design. The male ward is 32ft. by 24ft., and contains eight beds, giving 96 superficial feet to each bed, and is 13ft. high, or 1,248 cubic feet per bed. At the south end of this ward is an open fire with ventilating grate, for the purpose of distributing heated fresh air in winter and cold in summer. Behind the fire is a very large bay window, fitted all round with seats, and forming a delightful lounging place. The female ward contains six beds, and is 26ft. by 24ft. Great attention has been paid to sanitary requirements, and the ventilation and arrangements generally are very good.

On August 14 Lord Clinton opened the new Cottage Hospital at Ashburton. Lord Clinton arrived in Ashburton shortly before two o'clock, and drove to the Bull Ring, where he was met by a deputation representing the borough and the supporters of the hospital. A large party assembled at the old hospital, and walked thence in procession to the new building, a distance of half a mile. On the procession reaching the hospital grounds, the vicar, in his capacity as chairman of the Building Committee, handed to the president a silver-gilt key bearing the initial letters of the title of the hospital and the date. The hospital is a symmetrical one-storey building, consisting of two wings, with central entrance, constructed of a dark local limestone and Hamhill facings. It faces south, the windows on that side looking out upon a charming prospect of hill and dale. Each of the wings contains a large ward for four beds, a small single-bed ward, and a nurse's room; the east wing being intended for male patients, and the west for female. At the back of the building, opposite the entrance, is a large operating-room lighted from above. The bathroom, supplied with hot and cold water, is near at hand; and the wards are in electric communication. Kitchen and scullery, and other offices are well to the rear. The care of the hospital is to be entrusted to Miss Elliott, a trained nurse, who has had some years' experience in St. Thomas's Hospital, and the two hon. medical officers, Dr. Adams and Mr. H. Ubsdell, will continue their services.

Lady Tweeddale opened a new ward of Ross Memorial Hospital on August 7. The hospital is regarded as one of the most beneficent institutions of the district, thanks to the fostering care of the medical officers, Dr. Bruce and Dr. Adam; the lady superintendent, Miss Reid, of Balconie; and the head nurse, Miss Cross. The present is the second, if not the third, occasion on which the accommodation has had to be increased. The present enlargement consists of the extension of the eastern wing, which now forms a spacious and beautiful ward, containing six beds, bathroom, etc.; also the erection of a new wing to the north, in which provision is made for nurses' rooms. The work, designed by Mr. Joass, architect, has been excellently carried out. The Marchioness of Tweeddale was presented with a bouquet, and after pronouncing the ward open she inspected the whole building.

Dr. Drysdale is a bold man; he has actually tried to preach in Paris the evils of large families. Why, in France they give prizes to peasants with the largest families! But then their clergy are not married. A short time ago we received a circular for a clerical charity, which gave specimens of sad cases of numerous curates with twelve or thirteen children, and stipends of about £100 or £150 a year. But then common-sense says they hadn't ought; so we didn't subscribe to that charity. Dr. Drysdale said infant mortality in London was equal to 11 per 1,000 in wealthy districts and 38 per 1,000 in poor quarters. He deplored the fact that in the poorest districts of Paris—in Belleville, for instance—100 women had 175 children, while in the wealthy Champs Elysées quarter there were only 86 children for every 100 women. He concluded in favour of discouraging large families, but no resolution was submitted.

The work of the Durham Samaritan Society has been reviewed by a special commissioner, and the following notes made, which may be useful for other such societies:—(1) Irrespective of creed, all cases of sickness among the poor in the town (except infectious ones) are attended to when possible, when the aid of the nurses is applied for; (2) all that come under the care of the nurses are visited by some member of the committee; (3) patients, on leaving the hospital, have after-care given them when necessary; (4) convalescent home orders are given to both in-patients and out-patients when sea-air is recommended by the doctor; (5) artificial limbs and surgical appliances are supplied at cost price, the expense being defrayed, when possible, by the patients or their friends—in other cases by the society; (6) clothing is given, and railway fares paid, in cases of poverty; (7) nourishing food, wine, milk, etc., is supplied; (8) invalid appliances, necessary comforts, books, etc., are given or lent.

With what a shudder do we always read the heading "Buried Alive!" Many people have a fearful dread of such a fate, and an uneasy suspicion that such cases may be very numerous for aught we know. Quite recently a lady, residing at Dorbisch, Bohemia, where she owned considerable property, was buried, after a brief illness, in the family vault at the local cemetery. Four days afterwards her granddaughter was interred in the same place, but as the stone slab covering the aperture was raised the bystanders were horrified to see that the lid of the coffin below had been raised, and that the arm of the corpse was protruding. It was ascertained eventually that the unfortunate lady, who was supposed to have died of heart disease, had been buried alive. She had evidently recovered consciousness for a few minutes, and had found strength enough to burst open her coffin. The authorities are making enquiries into the case.

